# Case of Extreme Diffusion of Odours

**Published:** 1846-03

**Authors:** Alexander Wilcocks

**Affiliations:** Philadelphia


*Case of Extreme Diffusion of Odours.* By ALEXANDER WILCOCKS, M. D., of Philadelphia. (Communicated by Professor Dunglison.)

*Dear Sir*,—Your remarks last evening, upon the divisibility of odours, brought to my recollection a circumstance which I had nearly forgotten.

When at sea, two years ago, two hundred miles to the westward of the coast of Ireland, myself and many of the other passengers observed a smoky odour, which lasted during several successive days. An appeal was made to our captain for an explanation of the phenomenon. He informed us that he had frequently remarked it before, and that it was owing to the long continuance of easterly winds, which carried the odour of *burning peat* far out to sea.

Among the passengers was one gentleman, who, like myself, had lately listened to your lectures in the Jefferson Medical College. I need scarcely add, that the circumstance recalled to our minds the facts which you had adduced to prove this wonderful divisibility of odours.

A change in the wind, which soon occurred, carried our ship out of the latitude of the peat smoke.

With much respect, I am, dear sir,

Very truly yours,

ALEX. WILCOCKS.

Professor ROBLEY DUNGLISON.

*Philadelphia*, 28th January, 1846.

